# Unusual Mechanism of Facial Nerve Palsy Caused by Penetrating Neck Trauma

**DOI:** 10.1155/2020/1391692

**Published:** 2020-02-14

**Authors:** Marisa Klančnik, Petar Ivanišević, Marina Krnić Martinić, Petra Smoje, Marta Zrinka Vucemilovic

**Affiliations:** ^1^University of Split, University Hospital Center Split, Department of Otorhinolarynglogy, Spinčićeva 1, 21000 Split, Croatia; ^2^University of Split, School of Medicine, Šoltanska 2, 21000 Split, Croatia

## Abstract

We present a case of a low energy penetrating neck injury with only facial nerve (FN) palsy in the clinical finding. A 32-year-old male patient was admitted to the emergency department with a penetrating injury on the right side of the neck just behind the right ear, accompanied by evident right (FN) palsy, evaluated as House Brackmann grade IV. Computed tomography demonstrated an isolated soft tissue injury in the right retroauricular region without bone fracture, parotid gland lesion, or vascular structure involvement. The FN palsy was treated with corticosteroids (CS), and the patient had an uneventful and complete recovery. This case report presents an unusual mechanism of isolated, extratemporal, blunt injury of the FN after a penetrating neck injury followed by complete recovery.

## 1. Introduction

The facial nerve (FN) is mainly responsible for the facial movement, and its injury may cause great discomfort for the patient [1] as it may induce facial asymmetry, leading to a loss of balanced appearance and function. This may have a deleterious effect on a patient's psychology and social life [2].

The FN has a complex anatomical course, and dysfunction can be due to congenital, inflammatory, infectious, traumatic, and neoplastic etiologies [3], with trauma being the second most common cause of FN injury representing up to 15% of all cases [1, 4] The FN is also the second most commonly injured cranial nerve in blunt head trauma, after the olfactory [5].

The FN is frequently injured after head trauma with or without temporal bone fractures [6], but posttraumatic facial paralysis is often associated with high-energy trauma with facial bone fracture, especially the temporal bone and the base of the head [7, 8].

FN paralysis due to blunt trauma without imaging evidence of bony fracture has been reported only in few case reports [9], so the facial nerve paralysis after blunt trauma is considered very rare [1].

In this article, we present an unusual mechanism of a blunt injury to the extratemporal FN trunk following a sharp object, low energy, penetrating neck trauma that was treated conservatively and resulted in an uneventful and complete recovery of the function of the FN.

This case report was written following the CARE guidelines for case reports.

## 2. Case Report

A 32-year-old male patient was admitted to the emergency department with a penetrating injury on the right side of the neck, just behind and below the right ear, accompanied by evident weakness of the right side of his face.

The patient was a usually healthy man with no accompanying medical conditions; he was not taking any medication and reported no known allergies. This was a first accident he had in his workplace.

**Figure 1:**
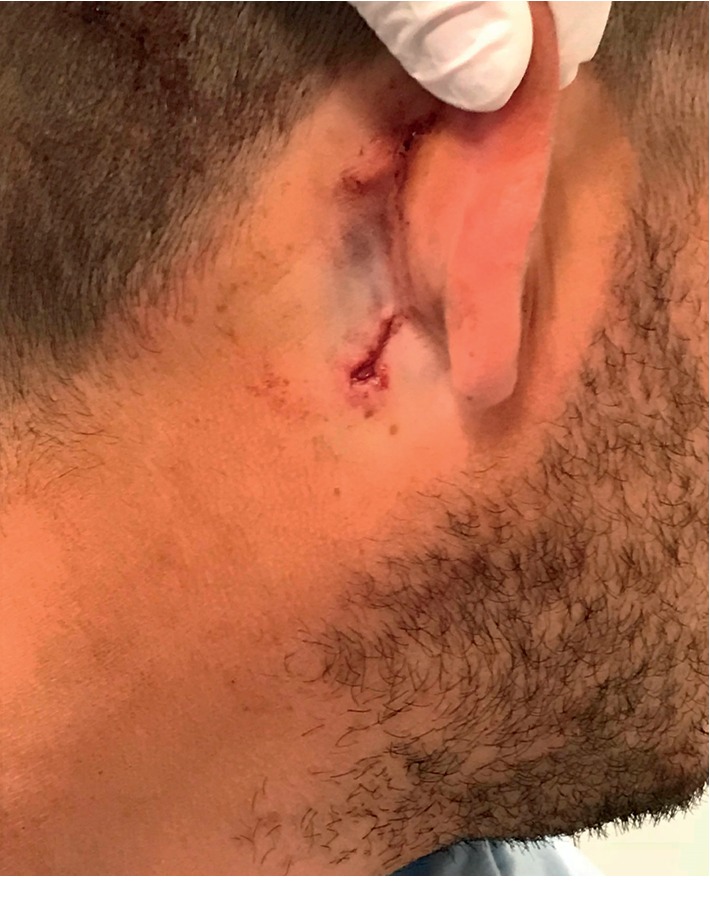
Retroauricular injury upon admission of the patient.

The patient acquired the injury when a construction protractor with a sharp tip fell from a height while he was working on a construction site. The protractor fell from a height of approximately two stories directly on his neck causing a small, penetrating injury behind and below his right ear as shown in Figure 1. After the impact, the patient described some bleeding from the injury site which stopped on compression.

**Figure 2:**
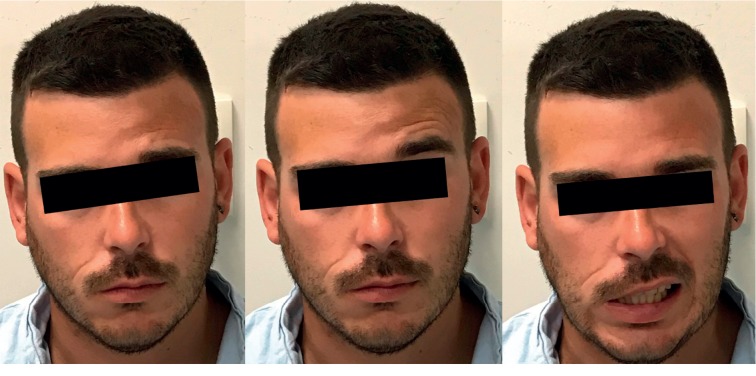
Peripheral FN palsy evaluated as HB grade 4 upon admission.

Upon admission, there were no signs of acute bleeding, his vital signs were in physiological ranges, and his neurological status was unchanged except the evident asymmetry of the face caused by incomplete, clinically peripheral right FN palsy evaluated as House Brackmann (HB) grade IV, as shown in Figure 2.

Otoscopic findings were normal bilaterally. A small retroauricular penetrating injury measuring 1 cm in diameter and 1 cm in depth was evident with mild edema of the right parotid region.

Laboratory blood analysis showed values of physiological ranges.

**Figure 3:**
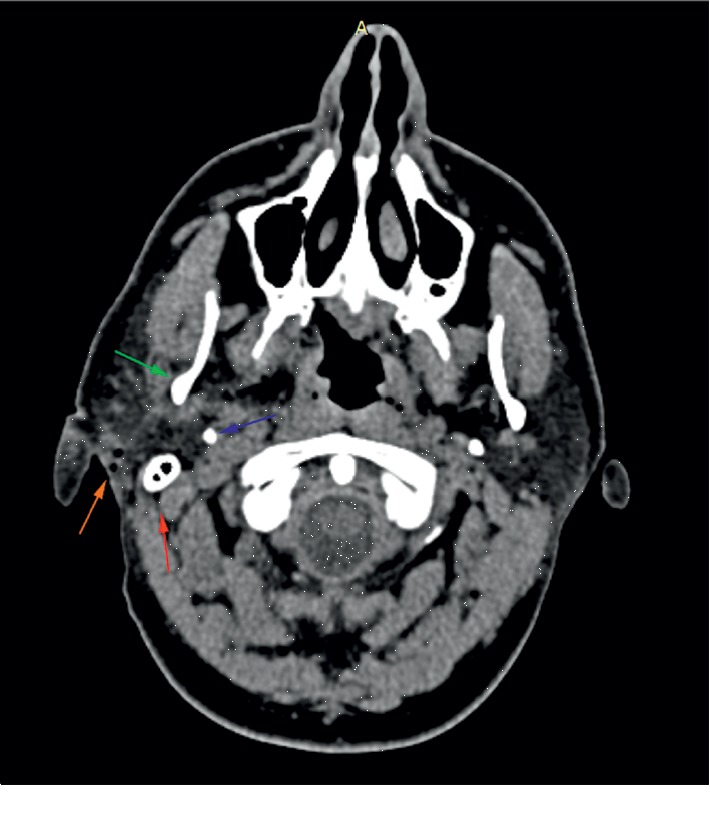
CT scan showing a mastoid tip (red arrow), styloid process (blue arrow), ramus of the mandible (green arrow), and entry trajectory (orange arrow).

An emergency soft tissue CT scan with contrast was performed, and a retroauricular subcutaneous gas collection measuring 0.6 cm was described, approximately 1.2 cm below the stylomastoid foramen, which corresponded the presumed sharp object entry trajectory. The right parotid showed normal morphology, without hematoma, and it showed no signs of bone of vascular trauma of the head or neck. CT scan that most closely presents the entry trajectory is presented in Figure 3.

The wound was copiously cleaned, a passive rubber drain was inserted, and the wound was sutured. The surgical management was limited to the skin and soft tissue. No formal attempt of FN exploration was conducted. The drain was removed 2 days later. There was no bleeding or edema after surgical management.

The patient received tetanus toxoid (TT) and tetanus immune globulin (TIG) vaccine. Additionally, dual antibiotic therapy (amoxicillin + clavulanic acid and metronidazole) was prescribed, and intravenous infusion of methylprednisolone was delivered in a tapering dosage schedule (250 mg daily for the initial 2 days, and over the next 10 days, was tapered by 40 mg every 2 days). Also, proton-pump inhibitors were administered for gastroprotection. Blood glucose levels were monitored simultaneously during the corticosteroid therapy, for which the levels varied between 4,8 and 6,9 mmmol/L.

Audiologic examination was conducted. Pure-tone audiometry verified normal hearing on both ears. Tympanometry showed type A tympanogram bilaterally. Acoustic stapedial reflex was intact bilaterally.

The mainstay of therapy was corticosteroid (SC) therapy which was given in a tapered manner for the following 14 days beginning with 2 mg/kg of methylprednisolone parenterally. The patient also received gastroprotective medication (proton-pump inhibitors) with the CS therapy.

The patient was released on the 7th day after injury with continuation of CS therapy in the form of tablets. The patient was engaged in physical therapy in the form of facial rehabilitation for acute facial palsy.

The patient attended regular ENT follow-up, first weekly for the first month and afterwards monthly. No additional medication was prescribed.

On ENT follow-up examination 6 months after the incident, his FN status was completely normal with complete restoration of the FN function evaluated as HB grade I. His recovery was otherwise uneventful.

## 3. Discussion

We present a case of a blunt injury of the FN trunk following a penetrating injury in the right retroauricular region of a young and otherwise healthy male. The incomplete FN palsy was treated conservatively, and surgical management was limited to soft tissue approximation and skin closure, and no attempt of FN exploration or surgical management was performed. The recommendations for surgical management are available only for FN palsy after temporal bone fracture or intratemporal blunt trauma, and those are not completely straightforward [10, 11]. There are no recommendations for surgical management of incomplete, extratemporal, blunt FN injury.

Our imaging studies were limited to soft tissue CT scan to exclude eventual parotid hematoma, vascular structure injury, or bone fracture. It is possible that MR imaging would produce additional information of soft tissue trauma [3], but as the mechanism of the injury seemed evident, we did not order additional imaging.

Our treatment of the FN palsy relied on tapered CS therapy reinforced with facial rehabilitation for acute FN palsy. The recently published study by Lee et al. [12] about FN injury after blunt craniofacial injury showed steroid therapy onset within 24 hours, and steroid therapeutic duration for longer than 14 days possessed a significantly better recovery rate. There was no conclusion about which doses of CS therapy should be administered and how should the doses be tapered. Our treatment was initialized in the fires 24 hours and lasted for 14 days which just reached the recommended cutoff.

Interestingly, in the study by Lee et al. [12], patients with penetrating injury to the neck were excluded. We found no studies with penetrating injury to the neck and consequently blunt or crush injury to the facial nerve which would guide the clinician toward a recommended treatment regimen.

We found only one similar case report describing a fish colliding with a diver with its jaw consequently lodging in the diver's retromandibular fossa [13]. The jaw was surgically removed, and the patient did not receive CS therapy. Initial palsy was described as complete, and the FN function was completely restored after 3 months.

Additionally, to the CS therapy, the patient engaged physical rehabilitation for acute FN palsy. It is mentioned in the literature that patients who experience facial palsy, irrespective of cause, can benefit from facial rehabilitation intervention [14] although the size of improvement gained by physical therapy is not jet clear in the setting of blunt peripheral FN injury.

Also, the time frame in which FN recovery should be expected regarding injury site, initial HB grade score, or injury mechanism is not clear. In this case, although the initial prognosis was good, it took 6 months for the patient to regain complete FN function.

This article showed a case of an extratemporal injury of the FN trunk caused by a penetrating injury to the neck which caused an incomplete FN palsy. Similar cases are rare in the literature, and there are no straightforward recommendations about imaging, surgical treatment, rehabilitation, or exact recommendations for medicamentous therapy, i.e. dosage, tapering, or duration of CS therapy for such FN injuries. Nevertheless, such injuries have favorable prognosis, and complete restoration of FN function, as in this case, can be expected.
